# Characterization of the complete chloroplast genome of *Pinus wangii* (Pinaceae), an endangered and endemic species in China

**DOI:** 10.1080/23802359.2018.1519381

**Published:** 2018-10-29

**Authors:** Mei-Qing Yang, Yan Du, Li-Zhen Ling

**Affiliations:** a School of Pharmacy, Baotou Medical College, Baotou, China;; b School of Biological Sciences and Technology, Liupanshui Normal University, Liupanshui, China

**Keywords:** Piuns wangii, Endangered species, chloroplast genome, SSR, phylogeny

## Abstract

The complete cp genome of *Piuns wangii* was 117,014 bp in size, containing a large single copy (LSC, 64,061 bp) region and a small single copy (SSC, 52,007 bp) region separated by two reduced inverted repeats (IRs, 473 bp). A total of 107 genes were predicted, including 73 protein-coding genes, 30 tRNA genes, and 4 rRNA genes. Most of the 51 simple sequence repeats (SSRs) were mononucleotides motifs of A/T types and were found to be located in non-coding regions. Phylogenetic analysis of Asian *Pinus* showed *P. wangii* clustered a strongly-supported clade with *Pinus morrisonicola*, *Pinus sibirica*, *Pinus wallichiana,* and *Pinus pumila*.

*Pinus wangii* Hu and W. C. Cheng is a five needle pine species, which belongs to the genus *Pinus* of the family Pinaceae. The species is sparsely distributed in limestone habitats of Xichou, Maguan, and Malipo counties of Yunnan Province (Fu et al. 1999). It is an important preferred afforestation tree species and wood sources for coffin, furniture, and potted landscapes (Xiang et al. 2017). In recent years, the natural habitat of *P. wangii* is severely damaged by over-exploitation, as a result, only five fragmented populations and 358 individuals are remaining on inaccessible cliffs (Zhou et al. 2012). It is the extremely small population species endemic to Yunnan province (Zhou et al. 2012) and ‘Endangered’ species in IUCN Red List (http://www.iucnredlist.org/). Meanwhile, it is listed as a national second-class protected plant in China (http://rep.iplant.cn/). It is urgent to take effective measures to protect this endangered and endemic species based on field investigation and scientific research (Zhang et al. 2015). In addition, its phylogenetic position is unclear in previous studies because of the lack of sampling. Here we characterized the complete chloroplast (cp) genome sequence of *P*. *wangii* based on the genome skimming sequencing data, detected the occurrence, type, and distribution of simple sequence repeats (SSRs) and constructed the phylogenetic tree based on the maximum-likelihood (ML) method.

The fresh leaves of *P. wangii* were collected from Kunming Botanical Garden. The voucher specimen (YMQ2018020) was deposited at Herbarium, Kunming Institute of Botany, CAS (KUN). The total DNA was extracted by TIANGEN Kit (Beijing, China). The genome skimming sequencing data was achieved on the Illumina Hiseq 2500 platform in Novogene Bioinformatics Technology Co., Ltd. (Beijing, China). The cp genome was assembled using CLC Genomics Workbench v.8.5.1 (CLC Bio, Aarhus, Denmark) with *P. armandii* Franch, as the reference (Accession Number KP412541) (Li et al. 2016). The annotation of the cp genome was conducted using Dual Organellar Genome Annotator (DOGMA) (Wyman et al. 2004) with manual adjustments for start and stop codons and intron/exon boundaries of protein-coding genes. The online tRNAscan-SE Search Service (Lowe and Chan 2016) (http://lowelab.ucsc.edu/tRNAscan-SE/) was used to further confirm tRNA genes. The complete cp genome sequence was submitted to the GenBank under Accession Number of MH167924. The circular map of the cp genome was drawn with the OGDRAW (Lohse et al. 2013; http://ogdraw.mpimp-golm.mpg.de/). The complete cp genome of *P. wangii* was 117,014 bp in length. It was the typical quadripartite structure, in which two inverted repeats (IRs, 473 bp) were separated by the large single-copy (LSC, 64,061 bp) and the small single-copy (SSC, 52,007 bp) regions. The cp genome had 107 genes, including 73 protein-coding genes (PCGs), 30 tRNA genes, and 4 rRNA genes. Most of genes occurred as single-copy, while three tRNAs (*trnS-GCU*, *trnR-ACG* and *trnT-GGU*) and one tRNA (*TrnI-GAU*) had two and three copies, respectively. In addition, two PCGs (*rps12* and *ycf3*) had two introns each, six PCGs (*atpF*, *petB*, *petD*, *rpl2*, *rpl16*, and *rpoC1*) and six tRNA genes (*trnA-UGC*, *trnG-UCC*, *trnI-GAU*, *trnK-UUU*, *trnL-UAA* and *trnV-UAC*) contained one intron. The overall GC content of the cp genome was 38.8%, while that of LSC, SSC and IR regions was 38.0%, 39.7% and 37.6%, respectively.

We used MISA (MicroSatellite identification tool; http://pgrc.ipk-gatersleben.de/misa/) to identify and localize the perfect microsatellites. The unit sizes of mono-, di-, tri-, tetra-, penta-, hexa-nucleotide repeats were set to minimum number of repeats of 10, 5, 4, 3, 3, 3, respectively. We removed one inverted repeat region (IRA) in SSRs analysis and manually checked the detected repeats. The occurrence and distribution of different types of SSR in the cp genome were summarized in Table 1. A total of 51 SSRs were detected throughout the cp genome of *P. wangii*, with 37, six, five, two and one for mono-, di-, tetra-, penta-, and hexa-nucleotide repeats, respectively. The majority of the mono-nucleotides were A or T and all of di-nucleotides were AT or TA repeats, which was consistent with the A/T-richness in the complete cp genome (Xuan et al. 2013). Five SSRs were located in four PCGs (*petD*, *rpl32*, *rpoB* and *ycf1* (×2)), four were observed in introns (*atpF*, *rps12* and *ycf3* (×2)), one was found in rRNA (rrn23) and one was identified in the boundary of IGS and PCG (*accD*) of the cp genome of *P. wangii*. Most of these SSRs were found in SSC region (28/51), followed by SSC region (23/51).

**Table 1.** Simple sequence repeats (SSRs) identified in the chloroplast genome of *Pinus wangii*.

		Location		Region			
Repeat	Number	LSC	SSC	CDS	rRNA	IGS	IGS-CDS
A/T	36	17	19	3		33	
C/G	1	1				1	
AT/AT	6	2	4	1		5	
AAAG/CTTT	1		1			1	
AAAT/ATTT	2	1	1			1	1
AATT/AATT	1	1				1	
ACCT/AGGT	1		1		1		
AAATC/ATTTG	1		1			1	
AAGGG/CCCTT	1	1				1	
ACCATC/ATGGTG	1		1	1			
Sum	51	23	28	5	1	44	1

LSC: large single-copy; SSC: small single-copy; CDS: coding sequence; IGS: intergenic space.

For maximum likelihood analyses, the complete chloroplast genome of 21 Asian *Pinus* species and one Picea species as outgroup were downloaded from NCBI GenBank. The combined datasets based on plastid genomes of 23 species were aligned by MAFFT v7.307 (Katoh and Standley 2013). A maximum-likelihood (ML) tree was constructed in RAxML (Stamatakis 2014) with the GTR + G model, and a total of 1000 bootstrap replicates were performed. The phylogenetic results showed that *P. wangii* formed a strongly-supported clade with *Pinus morrisonicola*, *Pinus sibirica*, *Pinus wallichiana* and *Pinus pumila* (Figure 1).

**Figure 1.**
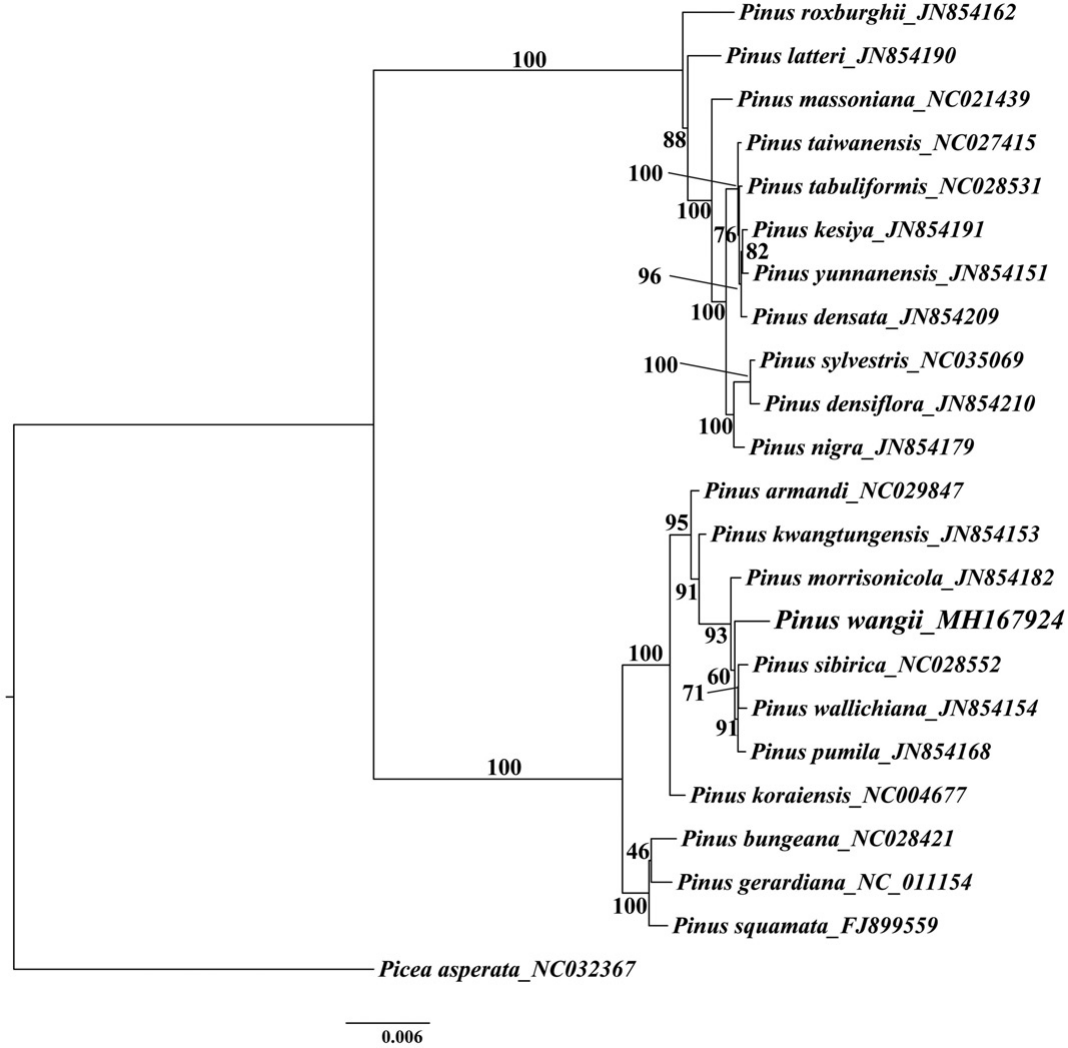
Phylogenetic tree inferred by Maximum Likelihood (ML) method based on the complete chloroplast genome of 22 species of Asian *Pinus*, bootstrap values (%) are shown on the branch.
